# 1737. Cost-Effectiveness Analysis of Catch-Up Vaccination with the 20-Valent Pneumococcal Conjugate Vaccine

**DOI:** 10.1093/ofid/ofad500.1568

**Published:** 2023-11-27

**Authors:** Mark Rozenbaum, Liping Huang, Johnna Perdrizet, Alejandro D Cane, Adriano Arguedas, Kyla hayford, Maria J Tort, Ruth Chapman, Vincenza Snow, Erica Chilson, Ray Farkouh, Desmond Dillon-Murphy

**Affiliations:** Pfizer Inc., Randstad, Noord-Holland, Netherlands; Pfizer Inc, Collegeville, Pennsylvania; Pfizer Inc, Collegeville, Pennsylvania; Pfizer, Collegeville, Pennsylvania; Pfizer, Collegeville, Pennsylvania; Pfizer, Collegeville, Pennsylvania; Pfizer Inc, Collegeville, Pennsylvania; Evidera, Inc, London, England, United Kingdom; Pfizer Vaccines, Collegeville, PA; Pfizer, Collegeville, Pennsylvania; Pfizer, Inc., New York, New York; Evidera PPD, London, England, United Kingdom

## Abstract

**Background:**

A 20-valent pneumococcal vaccine (PCV20) was approved in April 2023 by the US FDA for use in children < 18 years of age. Routine use in infants consists of a 3-dose schedule followed by a booster dose between 12 and 15 months of age. In addition, a catch-up program for children previously vaccinated with PCV13 who remain at risk of disease associated with the 7 new covered serotypes in PCV20 can potentially improve direct protection and accelerate the onset of indirect effects in the population. This study evaluated the cost-effectiveness of a PCV20 catch-up program in US children aged 14– 59 months who previously completed the recommended PCV13 series.

**Methods:**

A population-based, multi-cohort, decision-analytic Markov model was developed to estimate the public health impact and cost-effectiveness of a PCV20 catch-up program from both a societal (base-case) and health-care perspective over a 10-year time horizon. In the model a single supplemental dose was assumed to be given to 63% of children aged 14-59 months who had received a complete schedule of PCV13. Input parameters for costs, disutilities, and epidemiology of pneumonia and otitis media (OM) were extracted from published data sources. Data on invasive pneumococcal disease (IPD) were based on published and unpublished (ABC) data. PCV20 effectiveness estimates were based on PCV7/PCV13 studies.

**Results:**

The direct and indirect effects of a PCV20 catch-up program was estimated to prevent an additional 5,500 invasive IPD cases, 270,000 pneumonia cases, 0.6 million OM cases, and 1800 deaths compared to a 3+1 PCV20 program without a PCV20 catch-up program. This corresponds to a net gain of 30,000 life years or 55,600 QALYs. Most health gains were achieved due to the assumed accelerated indirect effects because of the catch-up program. Acquisition costs for the PCV20 catch-up program were offset by monetary savings from averted disease cases resulting in $800 million net saving. The catch-up program was also dominant from the health-care perspective.

**Conclusion:**

Implementation of a PCV20 catch-up program is expected to increase direct impact and accelerated indirect effects, more rapidly reducing pneumococcal disease burden and saving substantial health-care costs across the US population.

**Disclosures:**

**Mark Rozenbaum, Ph.D., M.B.A.**, Pfizer: Stocks/Bonds **Liping Huang, MD, MA, MS**, Pfizer Inc.: Stocks/Bonds **Johnna Perdrizet, MPH**, Pfizer Inc: Stocks/Bonds **Alejandro D. Cane, MD, PhD**, Pfizer: Stocks/Bonds **Adriano Arguedas, Medical director**, Pfizer: Emplyee|Pfizer: Stocks/Bonds **Kyla hayford, PhD**, Pfizer: Employee|Pfizer: Ownership Interest|Pfizer: Stocks/Bonds **Maria J Tort, PhD**, Pfizer Inc: Stocks/Bonds **Ruth Chapman, MSc, PhD**, Pfizer: Advisor/Consultant **Vincenza Snow, MD**, Pfizer Vaccines: employee|Pfizer Vaccines: Stocks/Bonds **Erica Chilson, PharmD**, Pfizer, Inc: Ownership Interest|Pfizer, Inc: Stocks/Bonds **Ray Farkouh, PhD**, Pfizer: Stocks/Bonds **Desmond Dillon-Murphy, MSc, PhD**, Pfizer: Advisor/Consultant

